# Multivariate Analysis as a Tool to Identify Concentrations from Strongly Overlapping Gas Spectra

**DOI:** 10.3390/s18051562

**Published:** 2018-05-15

**Authors:** Yannick Saalberg, Marcus Wolff

**Affiliations:** 1 Heinrich Blasius Institute of Physical Technologies, Hamburg University of Applied Sciences, Berliner Tor 21, 20099 Hamburg, Germany; yannick.saalberg@haw-hamburg.de; 2 School of Engineering and Computing, University of the West of Scotland, High Street, Paisley PA1 2BE, UK

**Keywords:** multivariate analysis, partial least squares regression, PLS, photoacoustic spectroscopy, PAS, OPO, overlapping spectra, concentration determination, VOC

## Abstract

We applied a multivariate analysis (MVA) to spectroscopic data of gas mixtures in the mid-IR in order to calculate the concentrations of the single components which exhibit strongly overlapping absorption spectra. This is a common challenge in broadband spectroscopy. Photoacoustic (PA) measurements of different volatile organic compounds (VOCs) in the wavelength region of 3250 nm to 3550 nm served as the exemplary detection technique. Partial least squares regression (PLS) was used to calculate concentrations from the PA spectra. After calibration, the PLS model was able to determine concentrations of single VOCs with a relative accuracy of 2.60%.

## 1. Introduction

**Figure 1:**
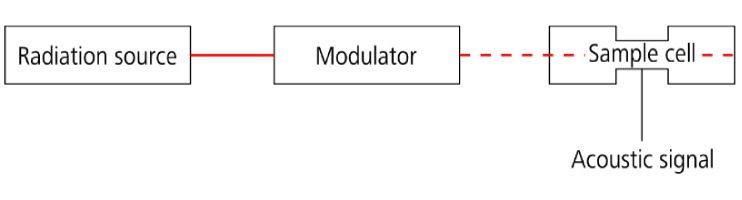
Schematic of a basic photoacoustic spectroscopy (PAS) setup.

Photoacoustic spectroscopy (PAS) is an established method for the detection of trace gases and the analysis of gaseous samples. PAS can be used for various applications such as stack gas emissions analysis, car exhaust gas analysis, explosives detection, control of manufacturing processes, and environmental control. It is based on the absorption of electromagnetic radiation. The thermal relaxation of the excited molecules leads to an increase in pressure. If the radiation is periodically modulated, it will result in an acoustic wave with the same frequency [1,2,3,4,5,6]. A schematic PAS setup is shown in Figure 1.

Spectroscopic sensors obtain their result by spectrum analysis. In practical applications, spectroscopic measurements are usually not performed on single component samples but on multicomponent mixtures. However, mixtures, especially of larger molecules, often result in strongly overlapping spectra [6,7]. In this case, an identification of the components and the determination of their concentrations are difficult as no wavelengths that are exclusively characteristic for single ingredients exist. However, in many situations, identification and concentration determination is possible with the tools of multivariate analysis (MVA). These techniques represent an alternative to traditional fitting methods [8,9] or machine learning methods such as artificial neural networks (ANN) or support vector machines [10,11]. MVA covers a large group of techniques. Their objective can be either [12]

- Classification of data or
- Multivariate regression.

Classification serves the purpose of discovering datasets with common properties. Regression, on the other hand, is supposed to identify relations between different sets of variables. The MVA is an established technique in different scientific areas such as psychology, sociology, environmental sciences, and biosciences [12,13].

We have developed the first spectroscopic sensor system for the six most relevant lung cancer biomarkers [14]. However, their spectra do not only feature single cross-sensitivities, but also cover the exact same wavelength range. Advancing our system to a real analyzer requires supplying it with a processing algorithm that allows deriving the ingredients’ concentrations of a mixture from a measured spectrum. This has now been accomplished.

This analytical task represents a regression problem and is very common in science [15]. Different regression methods are available in the field of multivariate analysis. Particularly common are the multiple linear regression (MLR), the principal component regression (PCR), and the partial least squares regression (PLS) [15,16]. When dealing with spectroscopic data, the PLS is superior to the other techniques because this data is strongly correlated and often noisy. Conversely, the MLR is more suitable for uncorrelated data and smaller sets [15].

A PLS model was trained with experimental spectra of single components and synthetic spectra of mixtures and tested on experimental mixture data. A reliable concentration determination could, for instance, lead to new methods in medical diagnostics [17]. However, the introduced multicomponent analysis might as well be applied to similar tasks such as environmental monitoring, the control of manufacturing processes, and explosives detection [1].

## 2. Materials and Methods

### 2.1. Experimental Setup

We developed a photoacoustic analyzer based on a continuous-wave optical parametric oscillator (cw-OPO) as the radiation source (Argos Model 2400-BB-5 Module C from Lockheed Martin Aculight Bothel, WA, USA). An ytterbium fiber laser represented the optical pump source (Model YLR-10-1064-LP-SF from IPG Photonics, Oxford, MA, USA). OPOs are spectrally tunable in a wide range, emitting relatively high powers. They use an optically nonlinear crystal to split a pump photon into two resulting photons called the signal and the idler, respectively. The idler wavelength can be tuned between 3250 nm and 3550 nm with output powers of up to 1.3 W. Details of the OPO and the pump laser can be obtained elsewhere [18].

The modulation of the radiation was realized by an all-optical Mach–Zehnder modulator. This in-house development is based on a Mach–Zehnder interferometer [19,20] with an optically nonlinear crystal inserted in one path (beam splitter BSW510 from Thorlabs, Newton, NJ, USA). The crystal’s refractive index can be changed by an electrical field (Pockels effect). A recombination of the two previously separated paths leads to constructive or destructive interference, depending on the applied electrical voltage.

A standard H-type cell sealed with calcium fluoride (CaF_2_) windows contained the sample. The name originates from the shape of the cell’s cross-section of which the center tube represents an acoustically resonant cavity. The resonator is flanked on both sides with buffer volumes of larger diameters. The length of the resonant cavity is 60 mm (diameter: 6 mm) and the overall length, including the buffer volumes, is 120 mm. The geometry of the cell is designed to result in a longitudinal resonance frequency of 2.81 kHz. It centrally incorporates the digital MEMS microphone for the detection of the photoacoustic signal (ICS-43432 from InvenSense, San Jose, CA, USA).

The 24 Bit I^2^S output signal of the MEMS microphone was processed by a microcontroller (PIC32 from Microchip Technology, Chandler, AZ, USA). The extraction of the photoacoustic signal at the modulation frequency was performed by means of a digital Goertzel filter [21,22,23]. In order to determine the current wavelength of the OPO radiation, a small fraction of the beam was directed to a spectrum analyzer (721A-IR from Bristol Instruments, Victor, NY, USA).

The obtained data, particularly the Goertzel filter result and the wavelength, were readout by a PC. At each wavelength, the photoacoustic signal was measured ten times, averaged in order to reduce noise, and divided by the averaged optical power.

For a single component, the photoacoustic signal S as function of the wavelength λ can be expressed as

(1) $$S\left(\lambda \right)=CP\left(\lambda \right)N_{tot}c\sigma \left(\lambda \right),$$

where C denotes the so-called *cell constant*, P the optical power, $N_{tot}$ the total molecular density, c the concentration of the absorbing substance, and σ its absorption cross-section [1,24,25]. The cell constant incorporates the cell geometry as well as the microphone position and the amplification due to the acoustic resonance. Since the emission power of the OPO is wavelength-dependent, we utilized the power normalized photoacoustic signal for further processing. The power is measured after passing through the sample cell by a thermal detector (3A-FS-SH from Ophir Optronics, Jerusalem, Israel). Reference spectra from Pacific Northwest National Library (PNNL) show a maximum absorption of 400 × 10^−6^ at a path length of one meter and a concentration of 1 ppm for the selected volatile organic compounds (VOCs) in the wavelength range of interest [26]. This corresponds to a power loss of approximately 0.45% after passage through the cell filled with 100 ppm VOC. Since the accuracy of the power meter is stated to be 3%, the power loss due to absorption was neglected for the normalization.

In multicomponent environments, the resulting spectrum is a linear combination of the single spectra. In the case of unsaturated absorption, the photoacoustic signal of an N-component gas mixture at a given wavelength is therefore given by [6,24,25]

(2) $$S\left(\lambda \right)=\overset{N}{\underset{i=1}{\sum }}S_{i}\left(\lambda \right)=CP\left(\lambda \right)N_{tot}\overset{N}{\underset{i=1}{\sum }}c_{i}\sigma_{i}\left(\lambda \right).$$

Further details of the experimental setup and the measuring technique can be found elsewhere [14].

### 2.2. Measurements

Gas samples of the following six VOCs were investigated with our experimental setup:

- 2-butanone,
- 1-propanol,
- isoprene,
- ethylbenzene,
- styrene, and
- hexanal.

These VOCs have all been linked to lung cancer as potential breath biomarkers. For an appropriate diagnostic application, a precise determination of the VOCs’ concentration is necessary. Single component gas samples of 100 ppm VOC in nitrogen (purity 5.0) were prepared in Tedlar bags [27,28]. The gas samples were created by evaporation of the liquid VOC inside the nitrogen-filled bag. The required liquid volume $V_{VOC}$ of a given VOC was calculated according to the following equation:

(3) $$V_{VOC}=\frac{V_{N_{2}}\cdot c_{VOC}\cdot M_{VOC}}{V_{ideal}\cdot \rho_{VOC}},$$

where $V_{N_{2}}$ denotes the volume of the nitrogen gas under normal pressure (the volume of the Tedlar bag), $c_{VOC}$ the desired VOC concentration, $M_{VOC}$ the molar mass of the VOC, $V_{ideal}$ the molar volume of an ideal gas, and $\rho_{VOC}$ the density of the liquid VOC. The liquid VOC was inserted into the Tedlar bag with a disposable syringe and left at room temperature for one hour. After this time, the liquid was completely evaporated. Although Tedlar bags do not completely prevent diffusion through the container wall, this effect can be neglected due to the relatively short storage time [27,28]. For multicomponent measurements, the required amounts of the different VOCs were inserted into the bag accordingly. The uncertainty of the concentrations equals ca. 1 ppm.

During the preparation time of the gas sample, the photoacoustic cell and the gas sampling system were evacuated in order to remove any potential residue from previous measurements.

The signal-to-noise ratios of our measurements are based on a reference noise measurement. During this measurement, the photoacoustic cell was filled with nitrogen (purity 5.0) which does not show any absorbance in the respective wavelength region. Since the maximum absorptions of the six VOCs differ, each VOC exhibits a specific signal-to-noise ratio. The values range from 43 dB to 55 dB.

### 2.3. Partial Least Squares Regression

The inner relation of the PLS can be resolved to

(4) $$Y=XB_{PLS}+G,$$

where $B_{PLS}$ corresponds to the so-called *regression coefficient* matrix, describing the relation between the datasets X (*predictor* matrix) and Y (*response* matrix), and G corresponds to the error matrix [15,16,29].

We performed the PLS calculations with MATLAB. This commercial software implements the Simple Partial Least Squares (SIMPLS) algorithm [30] which was used to determine the regression coefficient matrix $B_{PLS}$ in Equation (4). In this calibration process, also referred to as *training*, the photoacoustic reference spectra are the rows of the matrix X. The rows of Y consist of the associated (known) concentrations of the six VOCs. A high absolute value of a regression coefficient signalizes significant influence of a certain wavelength on the concentration [15].

After the model calibration, the concentrations vector Y of an unknown gas mixture can be determined by a simple vector (or matrix) multiplication of the photoacoustic spectrum vector X with the regression coefficient matrix $B_{PLS}$ according to Equation (4).

## 3. Results

### 3.1. Calibration of the Model

**Figure 2:**
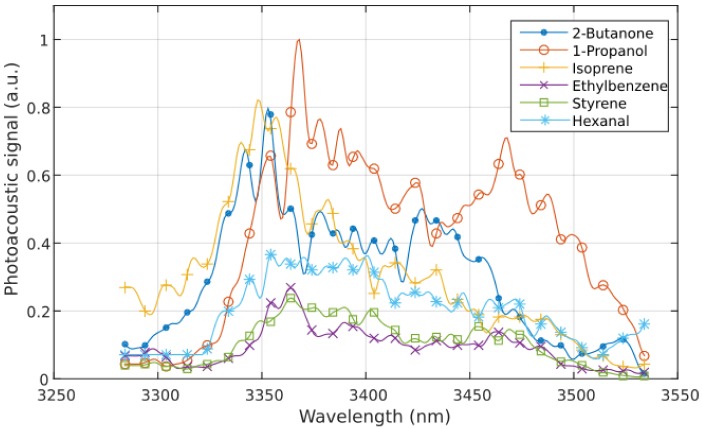
Measured volatile organic compound (VOC) spectra (each 100 ppm in nitrogen).

The PLS model was configured to calculate six principal components (PLS factors), each representing the contribution of one substance to the overall spectrum. In order to calculate the six variables, the number of reference spectra must be larger than six. The measured spectra of the six single VOCs in nitrogen (each 100 ppm) represented the first reference dataset. Each spectrum comprises the photoacoustic signals at 301 different wavelengths. The six spectra are shown in Figure 2. The photoacoustic signal is given in arbitrary units (a.u.) as the microphone outputs digital values.

**Figure 3:**
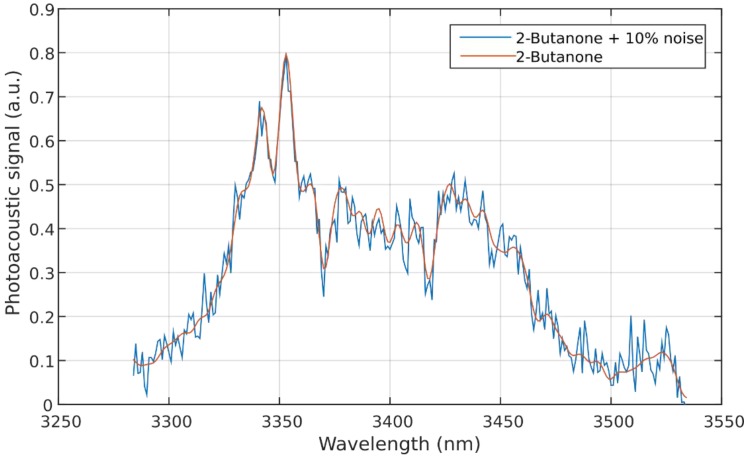
Spectra of 2-butanone with and without additional noise.

Additional reference spectra were synthetically generated by combining variations of the single VOC spectra. For this, we scaled the original measurements with the factors 0, 0.3, 0.7, or 1, and we added them to one another in all possible combinations. This resulted in a total of 4^6^ = 4096 reference spectra. The scaling “generated” concentrations of 0 ppm, 30 ppm, 70 ppm, and 100 ppm for each VOC. In order to consider the fluctuation of the photoacoustic signal and to improve the robustness of the model, Gaussian noise was added to the six original spectra. A long-term measurement of the photoacoustic signal at a constant wavelength resulted in an amplitude fluctuation of approximately 5%. Therefore, we decided on a noise power of 10% of the spectrum power, which is defined as the *root mean square* (*RMS*) of all measuring points. An example of spectra with and without additional noise is shown in Figure 3.

After the determination of the *regression coefficient matrix* using the SIMPLS algorithm, the calibration was evaluated. For this, a tenfold cross-validation was applied [15] to the reference dataset and reached a standard deviation of 2.65 ppm compared to the true concentrations without any *a priori* knowledge of the possible concentration levels. The model was able to explain 98% of the total variance, leaving only 2% being attributed to noise. In general, values above 90% are desirable in MVA. The *root mean square error of calibration (*RMSEC*)* between true concentrations $y_{i}$ and concentrations $\hat{y_{i}}$ calculated by the PLS model,

(5) $$RMSEC=\sqrt{\frac{1}{N}\overset{N}{\underset{i=0}{\sum }}{\left(y_{i}-\hat{y_{i}}\right)}^{2}},$$

reached 2.65 ppm [12]. After the training of the model, unknown mixture samples could be analyzed.

### 3.2. Multicomponent Measurements

**Figure 4:**
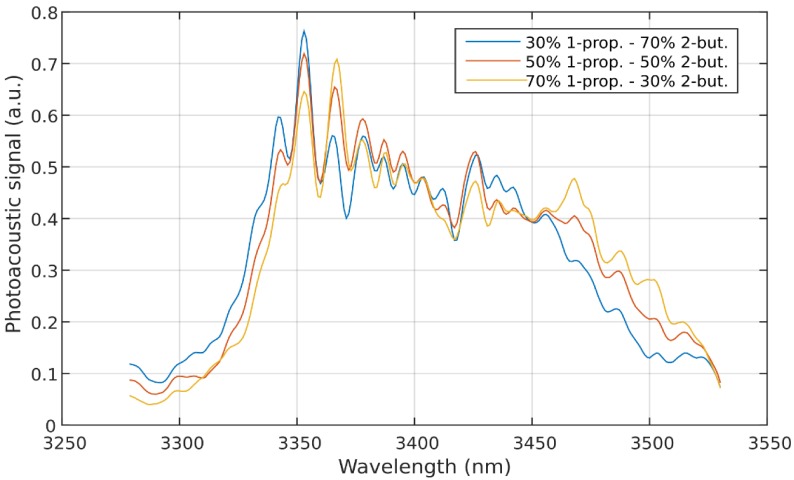
Photoacoustic spectra of 2-butanone/1-propanol mixtures.

The calibrated PLS model was applied to the photoacoustic spectra of two-component mixtures. For this, 2-butanone and 1-propanol were mixed (without the four other VOCs) in ratios of 30 ppm/70 ppm, 50 ppm/50 ppm, and 70 ppm/30 ppm, respectively. Figure 4 shows the three measurements. Table 1 lists the six concentrations determined by the trained PLS model.

The derived concentrations show a mean deviation from the true concentrations (sometimes referred to as *bias*) of approximately 5 × 10^−3^ ppm and a standard deviation of 3.93 ppm. The RMSE (Equation (5)) equals 3.82 ppm.

The accuracy of the results can be further improved if boundary conditions are taken into account. Concentrations of substances are logically limited to values larger or equal to zero. However, it is also possible to obtain negative concentrations as a result of the PLS. If negative concentrations are considered equal to zero, we increase the accuracy of the prediction. The resulting standard deviation of the concentration corresponds to 2.60 ppm and the RMSE to 2.79 ppm.

In order to assess the predictive ability of the model, the *predicted residual error sum of squares* (PRESS) was calculated. This value is strongly related to the standard deviation and the RMSE. The smaller the PRESS value, the better the model [29]. It can be calculated according to the following equation [12,31]:

(6) $$PRESS=\overset{N}{\underset{i=0}{\sum }}{\left(y_{i}-\hat{y_{i}}\right)}^{2},$$

where $y_{i}$ denotes the true concentrations (scaling factors) and $\hat{y_{i}}$ the values predicted by the model. For the standard and the enhanced dataset (negative concentrations = 0), the PRESS values equal 263 ppm^2^ and 140 ppm^2^, respectively.

## 4. Discussion

The application of multivariate analysis (MVA) in broadband spectroscopy leads to an increased specificity and sensitivity compared to sensors based on single wavelengths. Partial least squares regression (PLS) is a technique particularly suited for the analysis of spectroscopic data and allows for a fast and efficient processing. After the training of the model with spectra of known concentrations, the calculation of concentrations from new spectroscopic data represents a simple matrix multiplication. The computational effort is thus low compared to alternative methods like artificial neural networks, support vector machines, or traditional fitting. In addition, standard PLS algorithms like SIMPLS are often implemented in software such as MATLAB and can be applied without advanced coding skills.

The quality of the MVA results does not only depend on the applied method but also on the ingredients’ spectra. PLS would, for example, encounter problems if the single component spectra show a high resemblance to one another. In addition, the spectra need to be linearly independent. If the spectrum of one component is identical (or very similar) to a linear combination of two (or more) others, they would be indistinguishable. In summary, each one of the overlapping single spectra needs to provide characteristic features in the examined wavelength region.

The potential of MVA is limited by the quality of the experimental data. Spectra with features smaller than the noise cannot be resolved by the algorithm. This limitation strongly depends on the experimental setup, the absorption strength of the investigated components, as well as their concentrations. The influence of certain parameters that could deteriorate the performance was reduced. For instance, the normalization of the photoacoustic signal with respect to the optical power eliminates the effect of power fluctuations. Also, the OPO’s emission wavelength strongly depends on environmental parameters such as temperature. However, variations of the wavelength do not considerably affect our results because the wavelength steps are chosen to be comparably small (on average 0.2 nm).

We applied PLS to analyze the photoacoustic spectra of gaseous mixtures. The investigated volatile organic compounds 2-butanone, 1-propanol, isoprene, ethylbenzene, styrene, and hexanal exhibit strongly overlapping absorption spectra in the wavelength range of the optical parametric oscillator between 3250 nm to 3550 nm. After the calibration of the model with 4096 reference spectra (synthesized from the six original measurements), PLS was able to compute concentrations with a relative accuracy of 2.60%.

The results of this investigation were achieved using a comparably small number of measured reference spectra and validation spectra. More spectral data from different concentration levels and mixtures will be collected in the future in order to further improve and test the PLS model.

## Figures and Tables

**Table 1:** Concentrations of all six VOCs determined by the trained partial least squares (PLS) model.

VOC	30 ppm (2-but.)/70 ppm (1-prop.)	50 ppm (2-but.)/50 ppm (1-prop.)	70 ppm (2-but.)/30 ppm (1-prop.)
2-Butanone	33	46	71
1-Propanol	75	53	25
Isoprene	2	0	−3
Ethylbenzene	4	−1	−1
Styrene	−6	−2	4
Hexanal	−8	4	3
